# Proteomic Analysis Reveals Molecular Differences in the Development of Gastric Cancer

**DOI:** 10.1155/2022/8266544

**Published:** 2022-07-31

**Authors:** Rinchen Dhondrup, XiaoKang Zhang, Xuemei Feng, Dhondrup Lobsang, Qincuo Hua, Junli Liu, Ying Cuo, Sangji Zhuoma, Geri Duojie, Suonan Duojie Caidan, Samdrup Gyal

**Affiliations:** ^1^Tibetan Medical College of Qinghai University, Xining 810016, Qinghai, China; ^2^Jingjie PTM Bio (Hangzhou) Co.Ltd, Hangzhou 310018, Zhejiang, China; ^3^Qinghai Provincial Tibetan Hospital, Xining 810007, Qinghai, China; ^4^Affiliated Hospital of Qinghai University, Xining 810000, Qinghai, China

## Abstract

Gastric cancer (GC) is the 3rd leading cause of death from cancer and the 5th most common cancer worldwide. The detection rate of GC among Tibetans is significantly higher than that in Han Chinese, probably due to differences in their living habits, dietary structure, and environment. Despite such a high disease burden, the epidemiology of gastric cancer has not been studied in this population. Molecular markers are required to aid the diagnosis and treatment of GC. In this study, we collected gastric tissue samples from patients in Tibet with chronic nonatrophic gastritis (CNAG) (*n* = 6), chronic atrophic gastritis (CAG) (*n* = 7), gastric intraepithelial neoplasia (GIN) (*n* = 4), and GC (*n* = 5). The proteins in each group were analyzed using coupled label-free mass spectrometry. In addition, Gene Ontology (GO), Kyoto Encyclopedia of Genes and Genomes (KEGG) pathway enrichment, and protein interaction networks were used to analyze the differentially expressed proteins (DEPs) among groups. DEPs were quantified in comparisons of GC versus CNAG (223), GC versus GIN (100), and GIN versus CNAG (341). GO and KEGG analyses showed that the DEPs were mainly associated with immunity (GC versus CNAG) and cancer proliferation and metastasis (GC versus GIN, and GIN versus CNAG). Furthermore, the expression levels of cell proliferation and cytoskeleton-related proteins increased consistently during cancer development, such as ITGA4, DDC, and CPT1A; thus, they are potential diagnostic markers. These results obtained by proteomics analysis could improve our understanding of cancer biology in GC and provide a rich resource for data mining and discovering potential immunotherapy targets.

## 1. Introduction

GC is the fifth most common cancer and the third leading cause of cancer mortality globally, and it is a highly heterogeneous disease at the genetic and molecular levels [1, 2]. The number of GC cases in Asia accounts for more than half of the global cases, especially those in East Asia. The factors associated with GC include a high-salt diet, low intake of fruits and vegetables, smoking, and a family history of GC [3]. In addition, etiological factors, including a variety of genetic and epigenetic changes, are related to the GC process [4]. GC mainly develops through atrophic gastritis and intestinal metaplasia in a precancerous state. Cancer develops via a series of mucosal changes from nonatrophic gastritis to atrophic gastritis, intestinal metaplasia (IM), and GIN to GC [5, 6]. The prognosis of advanced GC is still very poor, but an early prognosis of GC can ensure long-term survival [7]. Precancerous lesions of gastric carcinoma comprise a class of GC that is closely related to changes in gastric mucosal pathology, with a key role in the progression of normal gastric mucosal cells into gastric cancerous cells [8]. It is not clear whether some or all of these lesions are directly involved in the development of GC. Early effective intervention is important for preventing and controlling GC [9]. Therefore, determining the relationship between premalignant lesions and the development of GC has important clinical significance for the early detection and treatment of GC.

Reliable biomarkers and potential therapeutic targets are highly desirable for understanding and treating GC, and they have been investigated widely [10]^.^ High-throughput omics techniques can be used to study the development of GC and the associated molecular mechanisms with unprecedented speed and in great detail [11]. Many previous studies of GC focused on the genome and transcriptome levels. GC-driven genes and abnormal regulatory pathways have been determined at the genome and transcription levels, thereby greatly improving our understanding of GC [12]. Genomic changes should be translated into changes at the protein level to affect the phenotype [13]. Proteomics can be used to study the characteristics of cells, tissues, or biological proteins, such as protein expression levels and posttranslational modifications [14]. Proteomics analysis has proven to be a convenient and effective method for discovering cancer biomarkers and therapeutic targets [15]. Planque et al. [16] identified five candidate lung cancer biomarkers by combining proteomic analysis of four lung cancer cell lines with informatics analysis of lung-related diseases. The combined proteomic and transcriptomic analysis provides a means of understanding gastric development and its relationship to GC occurrence [17]. Quantitative proteomics could be used for the accurate classification of triple-negative breast cancer (TNBC) subtypes [18]. Quantitative proteomics could help to identify the proteins related to drug resistance [19, 20].

Previous studies have analyzed tumor tissues from patients with colon [21], breast [22], and ovarian cancer [23] using mass spectrometry (MS) proteomics, and the results obtained in these studies can provide additional supplementary information for genomics research. In general, most studies of GC have focused on a single or small group of proteins or specific pathways [24–27].

These studies have greatly improved our understanding of GC, but proteins are highly dynamic and interactive. Large protein sets can regulate tissue growth through highly coordinated changes in their expression levels and play important roles in organ functioning [13]. Previous studies have identified lncRNA and mRNA that are differentially expressed between CAG and CNAG samples, providing useful information for identifying potential biomarkers for the diagnosis of CAG [28]; meanwhile, quantitative iTRAQ proteomics has shown that actin-binding proteins and Notch pathway-related proteins are differentially expressed between CAG and CNAG [14]. There has been extensive research on CNAG and CAG, but other processes involved in the development of GC need further research.

GIN is widely regarded as a precancerous lesion that should be closely followed or treated endoscopically [29, 30]. The prognosis of patients with gastric cancer can be greatly improved by early diagnosis and endoscopic resection of GIN [30]. However, it is barely known how protein expression patterns might differ and the molecular basis of different functions in the process of GC.

In the present study, we collected tissues from CNAG, CAG, GIN, and GC and determined DEPs using proteomics methods, as well as functional annotation by bioinformatics and disease association analysis. We aimed to determine the possible molecular regulation mechanisms involved in the occurrence and development of GC by identifying DEPs, as well as discovering candidate molecules for use as biomarkers.

## 2. Methods

### 2.1. Clinical Tissue Samples

Samples were obtained from patients with CAG, GC, CNAG, and GIN at Qinghai Provincial Tibetan Medical Hospital in Qinghai from January 2018 to December 2020. Subjects with any of the following medical histories were excluded: hypertension, diabetes, coronary heart disease, other tumors, radiotherapy, chemotherapy, or other drug therapy. Patients who met the requirements in combination with history, cytological examination, and pathological biopsy results were included. Five biopsies were obtained in accordance with the updated Sydney system [31], two biopsies each from the corpus and antrum, and a single biopsy from the angle of the stomach. The protocol was approved by Qinghai Provincial Tibetan Medical Hospital Research Ethics Committee. We collected written informed consent from all participating patients. Among the included 22 patients, 6 were diagnosed with CNAG, 7 were CAG, 4 were diagnosed with GIN, 5 were diagnosed with GC (Table 1and Supplementary Material 1). *Cancer* tissues were taken from the core area of the tumor, and we avoided including necrotic and adjacent noncancerous tissues. All samples were verified by pathologists at the hospital's pathology laboratory. All samples were rapidly frozen in liquid nitrogen and stored at −80°C for protein extraction.

### 2.2. Protein Extraction and Trypsin Digestion

**Figure 1:**
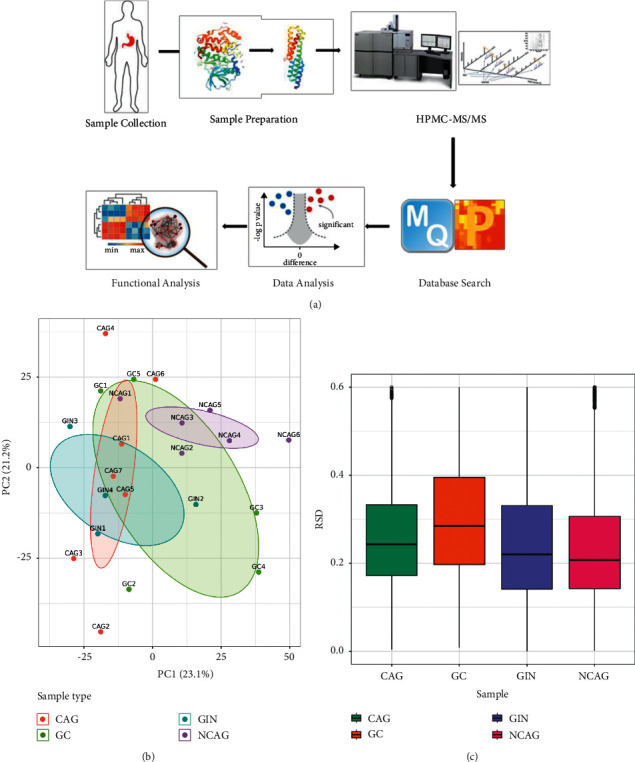
(a) General workflow of MS-based quantitative proteomics and bioinformatics analyses. (b) Two-dimensional scatter plot obtained by principal component analysis showing the distribution of all the quantified protein.samples. (c) Detection of the precision of proteins extracted from tissues.

Total proteins were extracted from the tissues as described previously by Li et al. [17]. Samples were minced and lysed in buffer (pH 8.0) containing 8 M urea, 100 mM Tris hydrochloride, and protease and phosphatase inhibitors (Thermo Fisher Scientific, Rockford, IL, USA). The tissue lysates were centrifuged for 10 min at 12000 × g and 4°C before collecting the supernatants to determine the protein concentration using a bicinchoninic acid protein assay kit (Pierce, Thermo Scientific, Germany). Next, approximately 100 *μ*g of protein per sample was reduced with 10 mM dithiothreitol (Sigma-Aldrich, St Louis, MO, USA) at 56°C before cooling the sample to room temperature and incubating with 20 mM iodoacetamide (Sigma-Aldrich, St Louis, MO, USA) in the dark for 30 min. The samples were digested with sequencing grade trypsin (Sigma-Aldrich, St Louis, MO, USA) for 24 h at 37°C, and all reactions were terminated with 10% (v/v) trifluoroacetic acid after digestion. Finally, the tryptic peptides were centrifuged to purify the peptides in C18 spin columns (Millipore, Waltham, MA, USA) with nine fractions using a stepwise increasing acetonitrile concentration gradient (6%, 9%, 12%, 15%, 18%, 21%, 25%, 30%, and 35%) under basic conditions (pH 10), before analyzing with liquid chromatography–MS/MS (LC-MS/MS). General workflow for functional proteomics analyses in CNAG, CAG, GIN, and GC (Figure 1(a)).

### 2.3. LC-MS/MS Analysis

LC-MS/MS analysis was performed as previously described [32,33]. Peptide samples were fractionated by high-pressure liquid chromatography (HPLC; Thermo EASY-nLC System, Waltham, MA, USA), where mobile phase A comprised 0.1% (v/v) formic acid in Milli-Q water and B comprised 0.1% formic acid in 100% acetonitrile. Peptides were eluted by HPLC with a mobile phase B gradient of 5–35% at a flow rate of 600 nL/min for 75 min. The samples were then analyzed with Orbitrap Fusion, Orbitrap Fusion Lumos, and Q Exactive Plus mass spectrometers (Thermo Fisher Scientific, Rockford, IL, USA) coupled to an EASY-nLC 1000 nanoflow LC system (Thermo Fisher Scientific, Rockford, IL, USA). MS/MS analysis was performed in the data-dependent mode. One full scan (300–1400 m/*z*, *R* = 60,000 at 200 m/*z*) was followed by up to 20 data-dependent MS/MS scans with higher-energy collision dissociation (target 2 × 10^3^ ions, maximum injection time 40 ms, isolation window 1.6 m/*z*, normalized collision energy of 27%).

### 2.4. Peptide Identification and Protein Quantification

The raw MS files were processed as described in a previous study [34]. Briefly, raw files were searched against the human National Center for Biotechnology Information (NCBI) Refseq protein database (*Homo*_sapiens_9606_SP_20201214. fasta, 20395 sequences) using Mascot 2.3 (Matrix Science Inc., Boston, MA, USA, version 2.2.1). The mass tolerance was set to 20 ppm for precursor ions, 50 ppm for product ions collected by QExactive HF, and 0.5 Da for product ions collected by Fusion. KR is a proteolytic cleavage site and it allows up to two missed cleavages. The database search engine set cystine carbamoyl methylation as a fixed modification and N-acetylation and methionine oxidation as variable modifications. In all of the identified peptides, peptide ions with a charge state of +1 or >4 and a different ratio for proteins of <2.0-fold or >0.5-fold were excluded, and the false discovery rate was adjusted to 1%. Intensity-based absolute quantification (iBAQ) was applied for protein quantification, and the iBAQ values were converted into iFOT values (fraction of total, iBAQ value of each protein divided by the sum of all iBAQ values of all proteins in the sample) as described previously [35]. The IFOT values were used to quantify low-abundance proteins. The false discovery rate for proteins was equal to the ratio of the number of assembled proteins from decoy database searches relative to the number of assembled proteins from target database searches.

### 2.5. MS Platform Quality Control (QC)

The trypsin in the tissue lysates was tested as a QC standard for MS. The QC standard was produced and operated using the same methods and conditions with the same software and GC parameters. We evaluated quantitative protein repeatability using three statistical analysis methods: Pearson's correlation coefficient, principal component analysis (PCA), and relative standard deviation.

### 2.6. Bioinformatics and Statistical Analysis

Limited selections were used to screen label-free quantitative data before DEPs analysis, as follows: (1) proteins with the same peptide found in two to three samples were included; (2) the protein identification confidence was set to 95%, and the false positive rate was less than 5% in the database; (3) the difference ratio of proteins was more than the 2.0-fold and the *p*-value was less than 0.05. Th DEPs were analyzed based on the GO secondary annotations. DEPs were classified using the GO database according to molecular function, cellular component, and biological process categories, and the significance of each protein function classification was determined using Fisher's exact test. To identify representative proteins in each tissue sample and determine their biological significance, we also conducted comparisons using the KEGG database (http://www.genome.jp/kegg/) to identify possibly enriched pathways. In addition, protein interaction network analysis and analyses of the similarities and differences in the DEPs between groups were conducted to identify the functional properties of the DEPs and their relevance to the research goal. For each category, a two-tailed Fisher's exact test was employed to test the enrichment of the DEPs against all identified proteins. The GO with a corrected *p*-value < 0.05 was considered significant. KEGG database was used to identify enriched pathways by a two-tailed Fisher's exact test to test the enrichment of the DEPs against all identified proteins. The pathway with a corrected *p*-value < 0.05 was considered significant. These pathways were classified into hierarchical categories according to the KEGG website. For further hierarchical clustering based on differentially expressed protein functional classification, we first collated all the categories obtained after enrichment along with their *p* values and then filtered for those categories which were at least enriched in one of the clusters with *p* value < 0.05. This filtered *p* value matrix was transformed by the function *x* = −log10 (*p* value). Finally these *x* values were z-transformed for each functional category. These *z* scores were then clustered by one-way hierarchical clustering (Euclidean distance, average linkage clustering) in Genesis. After comparing the DEPs in different groups with the STRING (v.11.0)(https://string-db.org) protein network interaction database, the interactions between the DEPs were obtained based on a confidence score >0.7 (high confidence). The protein-protein interactions (PPI) of dysregulated proteins predicted by STRING showed that most could interact with each other. STRING analysis showed that the DEPs formed strong networks with dynamic clusters.

## 3. Results

### 3.1. Protein Identification in Gastric Tissue Samples

**Figure 2:**
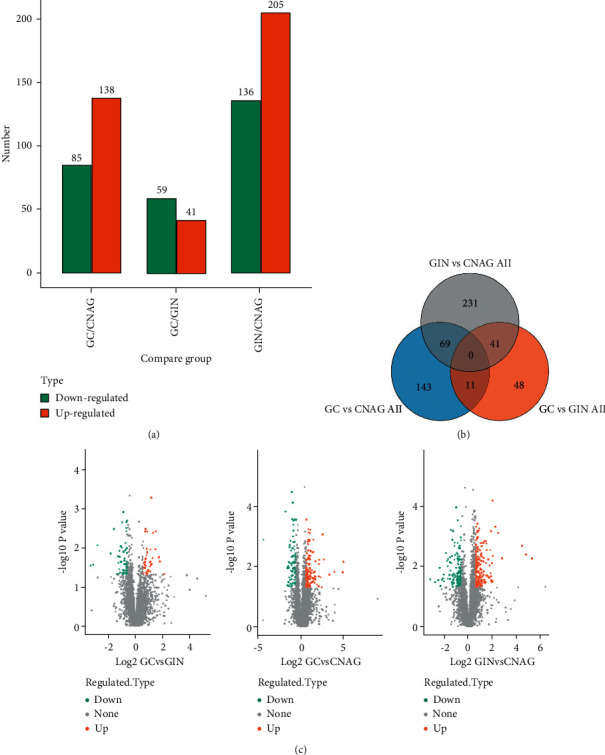
(a) Histogram showing the numbers of upregulated and downregulated differentially expressed proteins in different comparison. (b) Venn diagrams showing differences and similarities of the proteins identified in the three comparisons. The numbers of proteins represent those with more than twofold difference in expression in three comparisons, and the numbers of proteins shared in two or three cases. (c) Volcano plots showing the differentially expressed proteins determined in the comparisons of GC versus GIN, GC versus CNAG, and GIN versus CNAG.

We identified 100, 223, and 341 dysregulated proteins using Proteome Discoverer 1.4 in the comparisons of GC versus GIN, GC versus CNAG, and GIN versus CNAG, respectively (Figure 2). PCA and the Pearson's correlation coefficient indicated good quantitative repeatability (Figures 1(b) and 1(c)). We determined the expression levels of 100 proteins in GC versus GIN, where 41 proteins were upregulated, and 59 proteins were downregulated. The expression levels of 223 proteins were determined in GC versus CNAG, where 85 proteins were upregulated, and 138 proteins were downregulated. The expression levels of 341 proteins were determined in GIN versus CNAG, where 205 proteins were upregulated, and 136 proteins were downregulated (Figure 2 and Supplementary Material 2).

### 3.2. Subcellular Structural Localizations of DEPs

**Figure 3:**
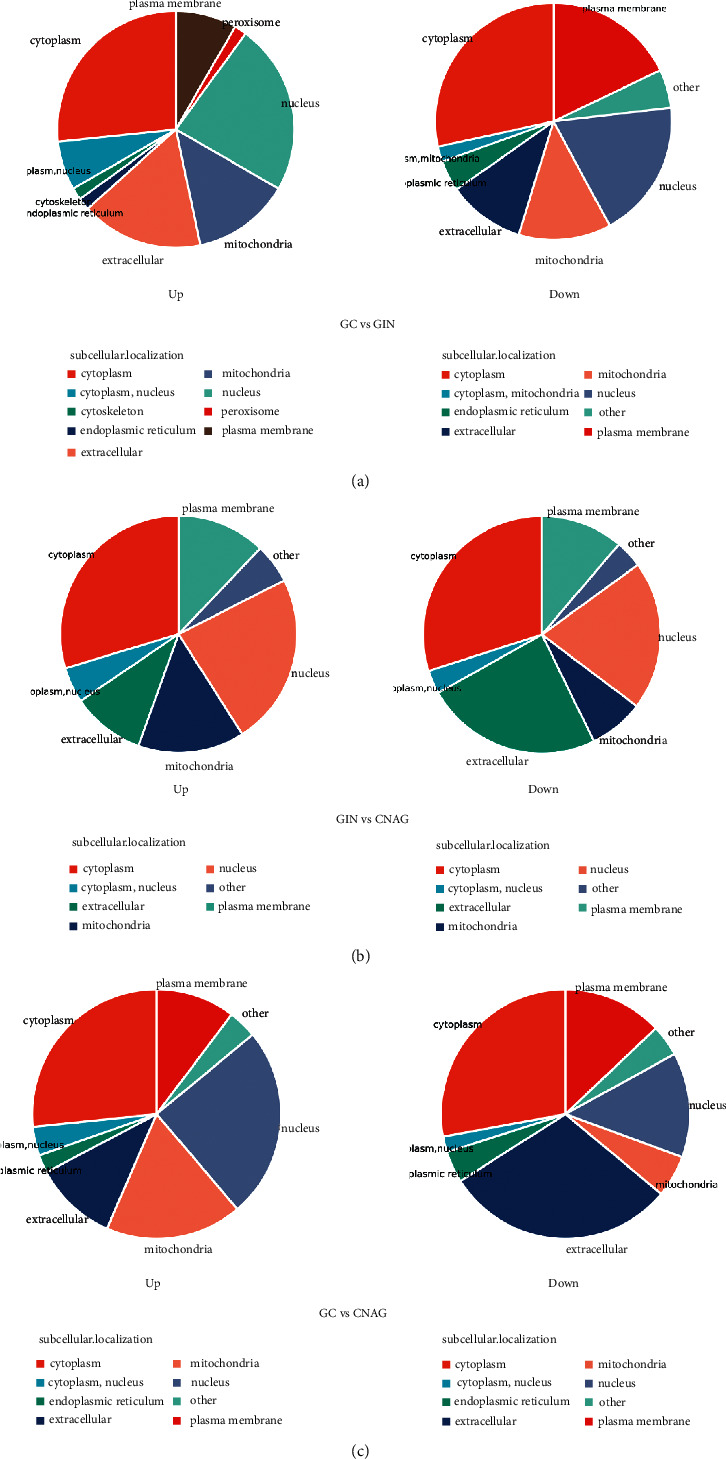
Subcellular localizations of differentially expressed proteins identified in comparisons of: (a) GC versus GIN; (b) GC versus CNAG; and (c) GIN versus CNAG.

The development of GC is strictly regulated by a series of signaling events and effectors. We conducted analyses to further investigate the functions of the DEPs. The subcellular localization indicated that the DEPs were annotated as cytoplasmic for GC versus GIN (Figure 3(a)). For GC versus CNAG, the results showed that the upregulated proteins were located in the cytoplasm and nucleus, and the downregulated proteins were located in the cytoplasm and extracellular areas (Figure 3(b)). For GIN versus CNAG, the upregulated proteins were annotated as located in the cytoplasm and nucleus, and the downregulated proteins were located in the cytoplasm, extracellular areas, and nucleus (Figure 3(c)). The cytoplasm is the main site for biochemical reactions. These results indicate that proteins in the cytoplasm and nucleus may play important roles in the development of GC.

### 3.3. Functional Annotations of DEPs Using GO

**Figure 4:**
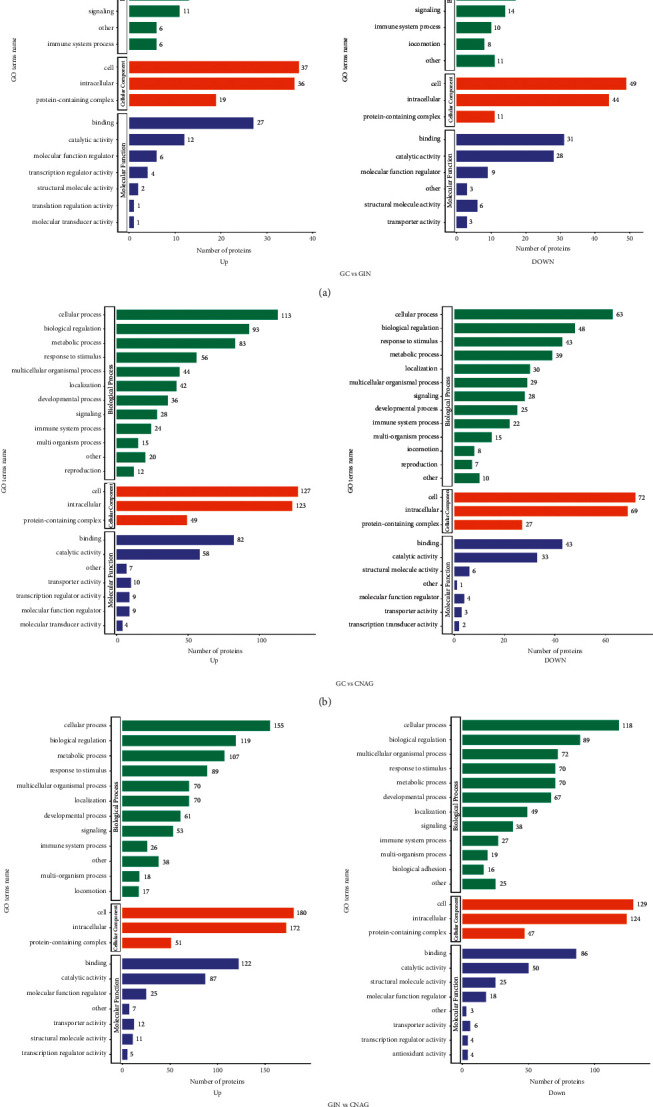
Classifications of the proteins identified using the GO database: (a) GC versus GIN; (b) GC versus CNAG; and (c) GIN versus CNAG.

**Figure 5:**
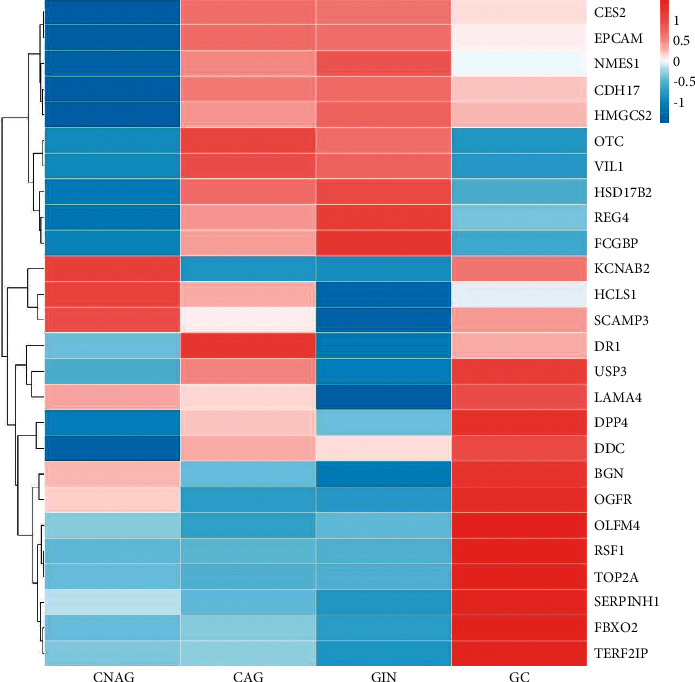
Clustered heatmap analysis of the differential proteins in CNAG, CAG, GIN, GC groups. Each column represents the protein information of one group of samples, and each row represents the relative expression level of each protein.

Furthermore, the DEPs quantified in each group were statistically analyzed based on GO primary annotations. The DEPs were classified into three categories (biological process, cellular component, and molecular function) based on GO annotations to assess the biological roles of the proteins from different perspectives. The GO enrichment analysis results for the DEPs were similar for GC versus GIN (Figure 4(a)), GC versus CNAG (Figure 4(b)), and GIN versus CNAG (Figure 4(c)), where they were mostly associated with a cellular process and biological regulation, and the molecular functions of these proteins were mainly related to catalytic activity and binding (Figure 5).

### 3.4. GO Enrichment Analysis

**Figure 6:**
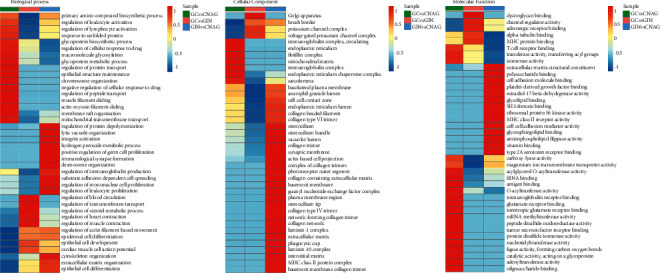
Biological process, cellular component, and molecular function analyses of the DEPs using the GO database.

For GC versus GIN, DEPs were mainly involved in the regulation of blood circulation and epithelial cell development in the biological process category, related to the Golgi apparatus in the cellular component category, and mainly related to T cell receptor binding and transferase activity in the molecular function category. For GC versus CNAG, DEPs were mainly involved in actin-myosin filament sliding and glycoprotein metabolic process in the biological process category, related to immunoglobulin complex, circulating and endoplasmic reticulum chaperone complex in the cellular component category, and mainly related to immunity and protein synthesis in the molecular function category. For GIN versus CNAG, the DEPs were related to substrate adhesion-dependent cell spreading and regulation of leukocyte proliferation in the biological process category, related to the phagocytic cup in the cellular component category, and mainly related to MHC class II receptor activity and cell-cell adhesion mediator activity in the molecular function category (Figure 6).

### 3.5. KEGG Pathway Enrichment Analysis

**Figure 7:**
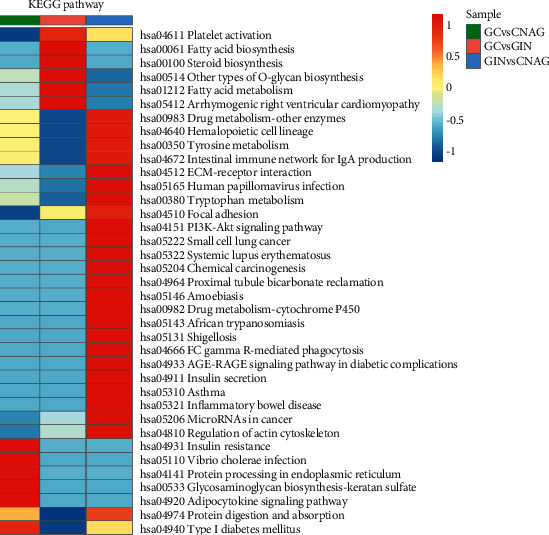
Classifications of proteins identified using the KEGG database.

KEGG enrichment analysis for GC versus GIN showed that the proteins were mainly involved in fatty acid metabolism and biosynthesis. For GC versus CNAG, the DEPs were enriched in aminoacyl-tRNA biosynthesis and insulin resistance. For GIN versus CNAG, the DEPs were enriched in intestinal immunity and inflammation, tryptophan metabolism, regulation of actin cytoskeleton, and extracellular matrix-receptor interaction (Figure 7).

### 3.6. Protein-Protein Interaction Network Analysis

**Figure 8:**
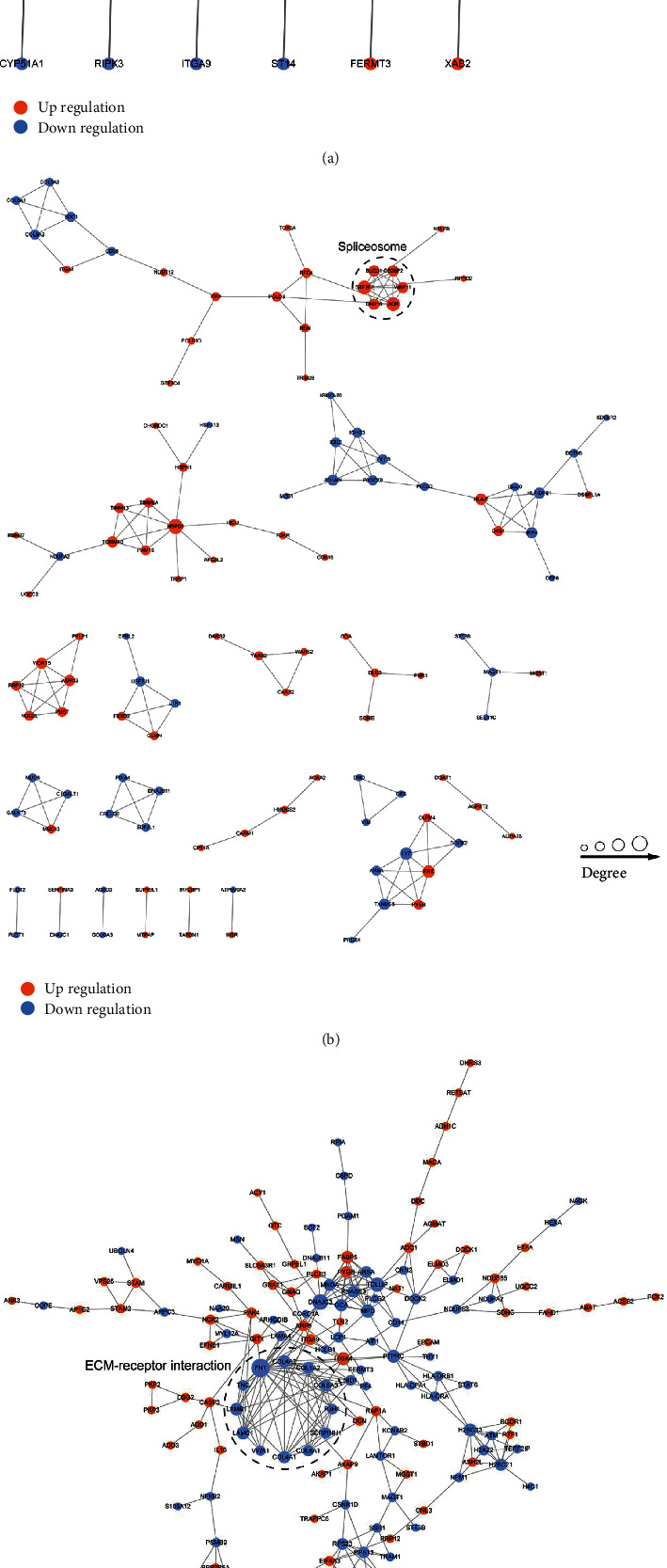
Association networks of dysregulated proteins: (a) protein-protein interaction network for GC versus GIN; (b) protein-protein interaction network for GC versus CNAG; and (c) protein-protein interaction network for GIN versus CNAG.

The cluster for GC versus GIN was mostly related to energy metabolism, cancer metastasis, and invasions, such as LAMTOR1 and TOM1 (Figure 8(a)). The cluster identified for GC versus CNAG contained proteins related to cell proliferation and migration, such as HSPD1, TOMM40, TIMM13, and TIMM8A. GTF2F2, RRP12, WDR75, GLMN, and WDR43 (Figure 8(b)). The cluster for GIN versus CNAG was mainly related to cell proliferation, cell migration, and invasions, such as PYGB, FABP5, ITGA4, ITGA9, RBX1, ARF6, PAK4, GIT1, and COMMD8 (Figure 8(c)).

### 3.7. Enrichment Clustering Analysis

**Figure 9:**
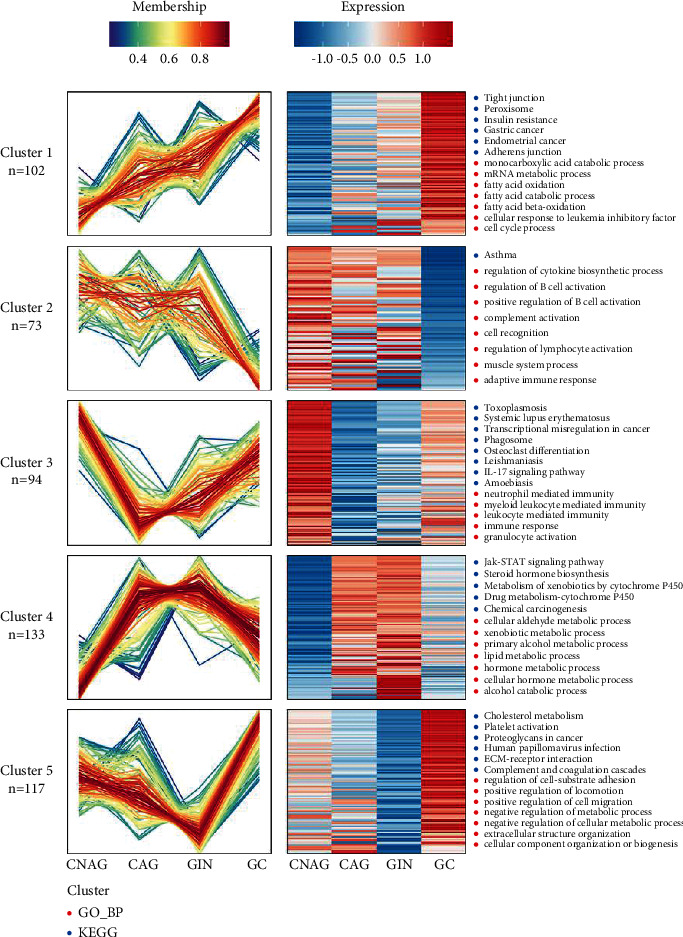
Proteins clustered and analyzed in GC, GIN, CNAG, and CAG according to biological processes with the GO and KEGG databases. Five clusters were determined by coexpression analysis. Left: coexpression patterns of the proteins in the five clusters. Right: representative GO terms for each cluster.

As shown in the clustered heatmap in Figure 9, the expression levels of proteins associated with cancer migration and gastric carcinogenesis were elevated during cancer development, where the signaling pathways for these proteins included gastric carcinogenesis and cancer migration. By contrast, the expression levels of immune and cytosolic factor-related proteins were decreased during cancer development, and these proteins were enriched in cellular pathways related to the complement system and immune system. According to our analysis, the expression levels of neutrophil-mediated, leukocyte-mediated, and immune-associated proteins were decreased from CNAG to CAG, whereas the expression levels of these proteins increased gradually during cancer progression. These immune-related proteins were mainly associated with transcriptional misregulation in cancer and the IL-17 signaling pathway. The expression levels of proteins related to lipid metabolism were higher in the GIN versus CNAG group than in the GC versus CNAG group, and these proteins were strongly associated with the Jak-STAT signaling pathway and steroid hormone biosynthesis. The expression levels of cancer migration-associated proteins were lowest in GIN, whereas the expression levels of these proteins were elevated during the progression from GIN to GC. These proteins activated pathways associated with complement and coagulation cascades and cancer migration.

## 4. Discussion

GC is one of the most common cancers throughout the world, and it has a high mortality rate (5). Early diagnostic screening and providing effective drug intervention targets are reliable methods for the detection and treatment of GC. However, the related molecules and regulatory mechanisms for GC are unclear, especially the key signaling pathways and optimum early markers and targets. In this study, we used proteomics to investigate gastric tissue samples collected from GC, GIN, CAG, and CNAG patients and identified DEPs related to GC to detect potentially important molecular and signaling networks, carcinogenic mechanisms, and specific biomarkers for GC diagnosis and treatment.

**Figure 10:**
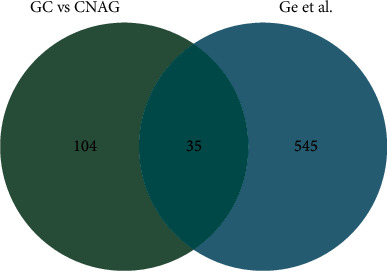
Venn diagrams showing the differences and similarities of differentially expressed proteins determined in the comparison of GC versus CNAG in our study, and nearby tissue-specific, and tumor-differentiated proteins analyzed by Ge et al.

CNAG is the most common type of chronic gastritis, and the risk of CAG is increased for patients with CNAG [36]. In this study, we identified and quantified a higher number of dysregulated proteins for GC versus CNAG. Further analysis showed that all of these dysregulated proteins had cancer-related associations, such as PDIA5 [37], DEF6 [38], MZB1 [39], TXNDC5 [40], YARS2 [41], MGST1 [42], and PIH1D1 [43]. Previous studies have shown that these proteins are associated with metastasis, invasion, proliferation, drug resistance, and a poor cancer prognosis. For GC versus CNAG, 36 DEPs were also related to specific tissues and tumors nearby, thereby indicating the reliability of our experimental results (9) (Figure 10). For the first time, our data analysis showed that WDR43 and WDR75 were associated with the development of GC. WD repetitive structural domains have biological functions via the epigenetic regulation of gene transcription, and the aberrant expression of WDR5 has been observed in various types of human cancers, including prostate cancer, breast cancer, and leukemia [44]. Previous studies have shown that WDR62 can be used as a diagnostic and prognostic biomarker for various cancers, and it is closely associated with infiltration by various immune cells [45]. For CNAG versus GC, KEGG analysis showed that aminoacyl-tRNA biosynthesis and insulin resistance were activated, thereby suggesting that the development of CNAG to GC may involve changes in adhesion proteins and cytoskeletal proteins. The expression levels of caveolin-1 and E-cadherin were significantly less in GC than in CNAG [46]. Protein-protein interaction analysis also showed that the protein interaction network for CAG involved proteins related to cell proliferation and migration, such as HSPD1, TOMM40, TIMM13, TIMM8A, GTF2F2, RRP12, WDR75, GLMN, and WDR43. Thus, the abnormal expression of proteins related to cell growth, proliferation, and migration may increase the likelihood of CNAG developing into GC.

For GIN versus GC, 100 DEPs were quantified, including SCAMP3 [47], USP3 [48], PIH1D1 [49], ACSF3 [50], INPP1 [51], VPS53 [52], and EPHA2 [53]. These proteins are associated with protein synthesis, the ubiquitinase system, cellular autophagy, and cancer migration, thereby suggesting that these proteins can interact with each other to control cell fates. Kocevar et al. [54] analysis of 30 different proteins with roles in GC development, including metabolism, development, death, cellular communication, and transport, also partially supported our results. KEGG analysis showed that the signaling pathways activated in GIN were related to the complement system, platelets, and autophagy, thereby suggesting that GIN involves inflammation and mucosal injury. The protein interaction network obtained between GIN and GC also involved LAMTOR1, and thus GIN may involve aberrant cellular autophagy.

For GIN versus CNAG, 341 DEPs were quantified by LC-MS/MS, and GO enrichment analysis showed that their molecular functions mainly included protein binding, the cellular components were mainly intracellular, and the biological processes mainly involved cellular processes and biological regulation. KEGG analysis identified roles for focal adhesion, the PI3K-Akt signaling pathway, and extracellular matrix−receptor interaction; thus, the development of GIN may involve abnormal cytoskeletal changes, cell proliferation, and migration. The protein interaction network included PYGB, FABP5, ITGA4, ITGA9, RBX1, ARF6, PAK4, GIT1, and COMMD8, which are associated with cell proliferation, cell migration, and invasion, thereby indicating that the expression of proteins associated with metastasis and invasion occurs during cancer development from CNAG to GIN.

To further understand the changes in protein expression from CNAG to CAG, GIN, and GC, we performed coexpression analysis, and the results showed that the expression levels of proteins associated with cancer migration and gastric carcinogenesis increased consistently during cancer formation and progression, such as ITGA4, DDC, and CPT1A. ITGA4 is an adhesion molecule that is actively involved in cellular extravasation [55]. Lymphovascular invasion (LVI) and nerve invasion (PNI) are two important pathological parameters, and ITGA4 is a reliable marker for the simultaneous detection and diagnosis of LVI and PNI, where it has been detected in colon, prostate, esophageal, lung, kidney, uterine, tongue, bladder, and liver cancers [56]. DDC is an enzyme involved in the biosynthetic pathway for the neurotransmitters dopamine and serotonin. DDC can be used to detect peritoneal micrometastases of GC with good sensitivity and specificity, especially for poorly differentiated adenocarcinomas [57]. The enzyme CPT1A resides in the outer mitochondrial membrane, and it catalyzes the reversible transfer of acyl groups between coenzyme A (CoA) and L-carnitine to convert acyl-CoA esters into acyl-carnitine esters [58]. CPT1A-mediated fatty acid oxidation promotes the metastasis of colorectal cancer cells by inhibiting anoikis [59]. Our findings also suggested that the expression levels of immune-related proteins were decreased, such as GMPR and HLA-DPB1. Lower expression of the HLA-DPB1 gene may lead to increased aggressive disease in adult adrenocortical tumors [60]. GMPR is closely associated with the formation of an invasive footprint, in vitro invasion, and the growth of melanoma cells [61]. The results obtained in previous studies combined with our findings indicate that the growth and invasion of cancer cells are important processes in GC formation and progression. Iuga et al. [32]suggested that upregulated proteins are more suitable as potential biomarkers than downregulated proteins during the development of GC. Therefore, we consider that the combination of ITGA4, DDC, and CPT1A could be used as potential diagnostic markers for GC. However, the value and utility of these protein molecules as potential biomarkers are still debatable and need to be fully validated.

In this study, we found that immunity, cell proliferation, and metastasis-related proteins may play important roles in the occurrence and progression of GC, and they are potential diagnostic markers for GC. Further studies are needed to verify whether these DEPs can be used as diagnostic markers for GC and whether they are targets for GC treatment.

## 5. Conclusion

Our findings provide a valuable resource for the early diagnosis and treatment of GC. Immunity, cell proliferation, and metastasis-related proteins related proteins are associated with the development and progression of GC. The DEPs were mainly associated with immunity (GC versus CNAG) and cancer proliferation and metastasis (GC versus GIN and GIN versus CNAG). ITGA4, DDC, and CPT1A are potentially diagnostic markers for GC.

## Figures and Tables

**Table 1:** Baseline characteristics of the patients included in this study.

Patient demographics	CNAG (*n* = 6)	CAG (*n* = 7)	GIN (*n* = 4)	GC (*n* = 5)
Age (years ± SD)	43.50 ± 12.48	54.71 ± 8.61	48.50 ± 13.17	57.2 ± 5.67
Sex (male, %)	66.66	71.42	75.00	80.00

## Data Availability

The data used to support the findings of the study are included within the article.

## Supplementary Materials

Supplementary Materials Additional file 1: Clinical characteristics and pathology of patients in this study. (Supplementary Materials (1)) Additional file 2: All upregulated (ratio ≥1.4) and downregulated proteins (ratio ≤0.714) in the comparisons of GC versus GIN, GC versus CNAG, and GIN versus CNAG are presented. (Supplementary Materials (2)).
