# Supplementary material for: Proteomic Analysis Reveals Molecular Differences in the Development of Gastric Cancer
Source: Evid Based Complement Alternat Med. 2022 Jul 31;2022:8266544. doi: 10.1155/2022/8266544 (PMC9357686; doi:10.1155/2022/8266544)
Supplement: Supplementary Materials — Additional file 1: Clinical characteristics and pathology of patients in this study. (Supplementary Materials (1)) Additional file 2: All upregulated (ratio ≥1.4) and downregulated proteins (ratio ≤0.714) in the comparisons of GC versus GIN, GC versus CNAG, and GIN versus CNAG are presented. (Supplementary Materials (2)). [file 8266544.f1.zip › 8266544.f1/Supplementary Materials 1 (1).pdf]

Table . Clinical characteristics and pathology of patients.

Gender	Atypia	Gastric Atrophy	Gastric intestinal metaplasia	Gastric cancer carcinogenesis
Female	-	+	+	-
Female	-	+	+	-
Male	-	+	+	-
Male	-	+	+	-
Male	-	+	+	-
Male	-	+	+	-
Male	-	+	+	-
Female	-	-	-	-
Male	-	-	-	-
Male	-	-	-	-
Male	-	-	-	-
Female	-	-	+	-
Male	-	-	+	-
Male	+	+	+	-
Male	+	-	+	-
Male	+	+	+	-
Female	+	+	+	-
Male	-	-	-	+
Male	-	+	+	+
Male	-	+	+	+
Female	-	-	+	+
Male	-	+	+	+

Note： - for negative, + for positive.
