# Supplementary material for: Proteomic Analysis Reveals Molecular Differences in the Development of Gastric Cancer
Source: Evid Based Complement Alternat Med. 2022 Jul 31;2022:8266544. doi: 10.1155/2022/8266544 (PMC9357686; doi:10.1155/2022/8266544)
Supplement: Supplementary Materials — Additional file 1: Clinical characteristics and pathology of patients in this study. (Supplementary Materials (1)) Additional file 2: All upregulated (ratio ≥1.4) and downregulated proteins (ratio ≤0.714) in the comparisons of GC versus GIN, GC versus CNAG, and GIN versus CNAG are presented. (Supplementary Materials (2)). [file 8266544.f1.zip › 8266544.f1/Supplementary Materials 2.pdf]

Protein descriptio	Gene name	GC/NCAG Ratio	GC/NCAG P value	Regulated Type	MW [kDa]	Coverage [%]	Peptides	Unique peptides
Unconventic	MYO1G	0.5123	0.0045623	Down	116.44	34.1	25	23
Immunoglob	--	0.3993	0.0019321	Down	22.83	47.7	12	3
AP-3 compl	AP3B1	0.7644	0.0006258	Down	121.32	35.9	32	32
Membrane-a	PGRMC1	1.343	2.31E-05	Up	21.671	44.1	10	9
Cocaine este	CES2	4.3025	0.0083086	Up	61.806	37	16	16
Pyridoxal ki	PDXK	0.7435	0.0111797	Down	35.102	45.2	12	12
Coatomer su	COPE	0.761	0.0042401	Down	34.482	59.4	11	11
AP-3 compl	AP3D1	0.7563	0.0138035	Down	130.16	38.2	33	33
Tumor prote	TP53I11	1.8111	0.002983	Up	21.054	18.5	4	4
Peripheral p	CASK	1.4935	0.0023118	Up	105.12	36.4	32	32
NPC intrace	NPC1	2.8047	0.0160081	Up	142.17	4.9	4	4
1-acyl-sn-gl	AGPAT2	4.2314	0.0462612	Up	30.914	9.7	2	2
Calpain-5 O	CAPN5	2.1529	0.0453875	Up	73.168	42.2	21	20
U4/U6.U5 tı	SART1	1.4619	0.0395907	Up	90.254	28.1	16	16
U4/U6 smal	PRPF3	1.3806	0.0440749	Up	77.528	15.1	8	8
ER membra	EMC8	1.3856	0.0442426	Up	23.773	45.7	7	7
Regulator o	RGS10	0.7649	0.0184345	Down	21.21	30.4	5	5
BUB3-intera	ZNF207	1.3155	0.0293878	Up	50.75	16.9	8	8
NADH dehy	NDUFA2	0.6194	0.0123457	Down	10.921	41.4	4	4
CD5 antiger	CD5L	0.3651	0.0336672	Down	38.087	49.3	11	11
Mitochondri	TIMM8A	1.58	0.0215677	Up	10.998	49.5	4	4
Pre-mRNA-	DHX16	2.0584	0.0007806	Up	119.26	13.9	11	10
RNA helicas	AQR	1.9006	0.0215537	Up	171.29	14.1	16	16
Protein O-G	OGA	1.8502	0.0014694	Up	102.91	15.2	9	9
Beta-1,4-gal	B4GALT4	0.4697	0.0207246	Down	40.041	26.7	7	7
General vesi	USO1	0.7518	0.0111217	Down	107.89	50.3	40	40
Nibrin OS=I	NBN	2.6968	0.003305	Up	84.958	13.1	6	6
NADH dehy	NDUFB1	0.6911	0.0276821	Down	6.9611	69	4	4
Diacylglyce	DGAT1	2.1788	0.0185317	Up	55.278	20.3	7	7
Flotillin-1 C	FLOT1	0.6654	0.0077379	Down	47.355	71	25	25
Kelch repea	KBTBD11	1.8818	0.0407114	Up	65.719	20.7	8	8
E3 ubiquitin	LTN1	0.6481	0.0421164	Down	200.55	10.7	13	13
Mitochondri	TOMM70	1.4685	0.0091726	Up	67.454	69.1	26	26
Erlin-2 OS=	ERLIN2	1.4298	0.0324149	Up	37.839	57.8	17	15
E3 ubiquitin	UBR5	2.7239	0.0012816	Up	309.35	4.9	9	9
Vesicle-assc	VAPB	1.423	0.0114467	Up	27.228	50.2	11	10
CD2 antiger	CD2BP2	2.0209	0.0227143	Up	37.646	23.5	5	5
Persulfide d	ETHE1	1.7104	0.0129253	Up	27.873	78	13	13
Double-strai	STAU1	1.4335	0.0273909	Up	63.182	37.6	19	19
Mitochondri	TOMM40	1.7873	0.0360006	Up	37.893	60.1	13	13
2'-5'-oligoad	OAS1	2.373	0.0228304	Up	46.028	27.8	9	9
Alpha-1-ant	SERPINA3	3.2711	0.0067155	Up	47.65	28.8	12	12
Immunoglob	JCHAIN	0.3774	0.0072413	Down	18.098	73.6	18	18
Immunoglob	IGKV3-20	0.5044	0.0199589	Down	12.557	40.5	4	3
Immunoglob	IGKC	0.5017	0.0048016	Down	11.765	100	16	2
Immunoglob	IGHG3	0.4092	0.0285513	Down	41.287	60.5	23	9
Immunoglob	IGHM	0.4265	0.0079275	Down	49.439	54.1	22	22
Immunoglob	IGHA1	0.395	0.0023092	Down	37.654	77.9	30	19
HLA class I	HLA-DPB1	0.4192	0.0385705	Down	29.159	26.4	6	5
Insulin recej	INSR	1.6675	0.0167768	Up	156.33	6.5	7	6

Beta-hexosa	HEXA	0.7136	0.0344754	Down	60.702	31.2	15	15
Protein disu	P4HB	0.7482	0.0179742	Down	57.116	77.4	41	41
Calpain-1 ca	CAPN1	0.76	0.0072914	Down	81.889	47.6	34	34
Heat shock	HSP90AA1	1.3537	0.0230457	Up	84.659	55.5	48	28
Signal recog	SRPRA	0.7648	0.0200655	Down	69.81	33.7	18	18
Monocyte d	CD14	0.7438	0.0176744	Down	40.076	42.9	10	10
Vimentin OS	VIM	0.6544	0.0121271	Down	53.651	78.3	46	37
Immunoglol	IGLC3	0.4803	0.0438534	Down	11.265	96.2	11	2
DNA-directe	POLR1D	2.0355	0.0112702	Up	15.237	61.7	6	6
Microsomal	MGST1	1.8526	0.0007491	Up	17.598	34.8	7	7
60 kDa heat	HSPD1	1.5372	0.0376263	Up	61.054	63.4	38	38
Endoplasmi	HSPA5	0.7226	0.004711	Down	72.332	57.6	42	41
Solute carrie	SLC2A1	1.7256	0.0184124	Up	54.083	13.8	7	7
Glycogen pl	PYGB	1.6386	0.0167044	Up	96.695	57.8	48	39
Ras-related	RALA	1.3986	0.0016431	Up	23.567	44.2	9	6
DNA topois	TOP2A	15.1146	0.0152266	Up	174.38	18.5	23	17
Dystrophin	DMD	0.5124	0.0363481	Down	426.74	23.6	63	61
Collagen alp	COL6A1	0.4266	0.0014078	Down	108.53	34	32	32
Collagen alp	COL6A2	0.4449	0.0029973	Down	108.58	29.4	30	30
Collagen alp	COL6A3	0.5056	0.0076789	Down	343.67	42.8	119	119
Integrin alpl	ITGA4	2.2636	0.0031805	Up	114.9	4.9	4	4
Protein disu	PDIA4	0.622	0.0002801	Down	72.932	59.1	46	45
HLA class I	HLA-E	1.6542	0.0059284	Up	40.057	36.9	11	8
Glycogen [s	GYS1	0.7438	0.0152867	Down	83.785	27	15	15
General tran	GTF2F2	1.6089	0.0255824	Up	28.38	30.1	6	6
Endoplasmi	HSP90B1	0.7234	0.0018836	Down	92.468	69.5	65	53
Arylsulfatas	ARSA	0.5466	0.0116831	Down	53.588	28.6	8	8
CD44 antige	CD44	0.7113	0.0341556	Down	81.537	13.1	8	8
Epithelial ce	EPCAM	3.1042	0.0213812	Up	34.932	36.9	12	12
1-phosphati	PLCG2	0.4645	0.0091439	Down	147.87	32.8	30	30
Desmin OS=	DES	0.4426	0.0378208	Down	53.535	68.7	36	30
Regulator of	RCC1	1.3444	0.0249496	Up	44.969	57	14	14
Syndecan-1	SDC1	0.5751	0.0365048	Down	32.461	15.8	5	5
Aromatic-L-	DDC	5.417	0.0497939	Up	53.926	17.3	7	7
Voltage-dep	VDAC1	1.3875	0.0044468	Up	30.772	83.4	21	21
NADPH:adr	FDXR	1.9743	0.046206	Up	53.836	40.9	15	15
Peptidyl-pro	PPIB	0.6888	0.004729	Down	23.742	61.6	18	18
Peptidyl-pro	FKBP2	0.5853	0.002789	Down	15.649	64.1	10	10
Dipeptidyl p	DPP4	5.8121	0.0008624	Up	88.278	31.1	20	20
ADP-ribosy	CD38	0.5422	0.010879	Down	34.328	43	14	14
Sorcin OS=]	SRI	1.4089	0.0014446	Up	21.676	46.5	8	8
ER lumen pr	KDELR2	0.617	0.0233174	Down	24.422	24.5	4	2
Heat shock	HSPA4	1.3196	0.0098266	Up	94.33	70.1	51	47
Carbonic an	CA8	0.651	0.0370959	Down	32.973	33.4	6	6
Replication	RFC4	1.7514	0.0472456	Up	39.681	24.2	7	7
Glutaredoxi	GLRX	0.6123	0.0061678	Down	11.776	50.9	6	6
Serine/threo	PPP1CC	1.3424	0.0159413	Up	36.983	51.7	19	2
Serpin B5 C	SERPINB5	2.695	0.03046	Up	42.1	70.4	21	21
Dolichyl-dip	DDOST	0.733	0.0098212	Down	50.8	53.7	18	18
Protein BUI	BUD31	1.5409	0.0433055	Up	17	54.2	9	9
Protein kina	PRKCI	1.3408	0.0469894	Up	68.262	19.1	9	9
Lamina-asso	TMPO	1.4216	0.0293579	Up	75.491	57.9	31	20

Tyrosine-pro	FRK	1.6993	0.0368391	Up	58.253	13.3	6	5
Leucine-rich	LRPPRC	1.3724	0.0197444	Up	157.9	61.6	79	78
3-ketoacyl-C	ACAA2	1.8185	0.0056951	Up	41.924	77.3	24	24
Glucosamine	GNPDA1	1.3641	0.0190516	Up	32.668	68.9	14	9
Lanosterol s	LSS	0.4961	0.0150219	Down	83.308	42.6	28	28
T-complex p	CCT5	1.3492	0.0259548	Up	59.67	62.3	34	34
Heat shock 7	HSPA13	0.4593	0.0266525	Down	51.927	22.1	10	10
Casein kinas	CSNK1D	0.7198	0.025174	Down	47.33	18.6	7	7
Protein ERG	LMAN1	0.6725	0.0056461	Down	57.548	44.1	22	22
Cysteine--tR	CARS1	0.7571	0.0046233	Down	85.472	45.1	25	25
Carnitine O-	CPT1A	3.1107	0.0151336	Up	88.367	46.6	37	37
Hsc70-intera	ST13	1.3134	0.0076193	Up	41.331	35	13	13
B-cell recep	BCAP31	1.6662	0.0036332	Up	27.991	45.9	17	17
Peroxisomal	HSD17B4	0.6959	0.0450975	Down	79.685	62.9	35	35
Arfaptin-2 C	ARFIP2	0.7022	0.0162622	Down	37.855	34.6	9	9
Hydroxymet	HMGCS2	3.2725	0.0224483	Up	56.635	52.4	21	20
Exportin-2 C	CSE1L	1.4685	0.0402031	Up	110.42	43.6	38	38
Mesencepha	MANF	0.6729	0.0062637	Down	20.7	60.4	18	18
Peroxisomal	PEX3	1.4687	0.0158965	Up	42.139	13.9	4	4
Synaptojani	SYNJ2BP	0.6709	0.007586	Down	15.928	17.9	2	2
Protein FAM	FAM3B	0.4535	0.0388862	Down	25.982	45.5	9	9
Protein trans	SEC61B	0.727	0.0024042	Down	9.9743	37.5	3	3
10 kDa heat	HSPE1	1.4828	0.002714	Up	10.932	81.4	14	14
Lysozyme C	LYZ	0.5202	0.0118605	Down	16.537	68.9	12	12
AP-1 compl	AP1S1	0.7453	0.0388405	Down	18.733	44.3	7	5
Nucleobindi	NUCB2	0.5354	0.0015516	Down	50.222	61.9	26	24
Vigilin OS=	HDLBP	0.7275	0.0049394	Down	141.45	48.3	57	57
Peptidyl-pro	FKBP4	1.4818	0.0276009	Up	51.804	73.6	33	33
Galectin-10	CLC	0.3392	0.0433197	Down	16.453	33.8	4	4
3-ketodihyd	KDSR	1.4134	0.0163974	Up	36.187	8.1	2	2
Regeneratin	REG3A	0.0475	0.0012873	Down	19.395	41.7	8	8
Synaptic fur	FMR1	1.6007	0.0059916	Up	71.174	17.2	8	5
Protocadher	PCDH1	1.7765	0.0244825	Up	114.74	12	9	9
Golgin subfa	GOLGA3	0.6477	0.0114666	Down	167.35	41.8	49	49
Golgin subfa	GOLGA2	0.7368	0.0079677	Down	113.08	45.6	33	33
Polypeptide	GALNT1	0.7137	0.0374332	Down	64.218	41	18	18
Cadherin-17	CDH17	29.8343	0.0157386	Up	92.218	54.4	36	36
Vesicular in	LMAN2	0.6806	0.0009063	Down	40.228	59.8	20	20
Epidermal g	EPS8	1.7918	0.012799	Up	91.88	35.8	21	21
Heat shock p	TRAP1	1.9331	0.0052643	Up	80.109	39.3	24	24
Peroxiredox	PRDX4	0.5989	0.0015226	Down	30.54	66.8	19	15
DnaJ homol	DNAJC3	0.711	0.0342504	Down	57.579	39.9	19	19
Transcriptio	TRIM28	1.5228	0.0190925	Up	88.549	60.8	32	32
Polyadenyla	PABPC4	0.7176	0.0134528	Down	70.782	33.2	23	16
Microfibrilla	MFAP5	0.5687	0.0121293	Down	19.611	27.7	6	6
Disintegrin a	ADAM9	1.4906	0.0430124	Up	90.555	10.3	7	7
Mothers aga	SMAD4	1.3804	0.0203636	Up	60.438	18.1	8	8
Cullin-4B O	CUL4B	1.4451	0.0498936	Up	103.98	28.6	22	14
Protein scrib	SCRIB	1.5742	0.0469684	Up	174.88	16.3	17	15
Flotillin-2 C	FLOT2	0.6652	0.0180362	Down	47.064	47.9	18	18
Polypeptide	GALNT3	0.5993	0.0164708	Down	72.609	39.5	19	17
Protein disu	PDIA5	0.5117	7.567E-05	Down	59.594	54.9	28	28

Delta(14)-st	LBR	1.4067	0.0229133	Up	70.702	18.2	10	10
Chromodom	CHD4	1.3673	0.0245381	Up	218	29.2	42	33
Signal pepti	SPCS2	0.6793	0.043288	Down	25.003	50.9	10	9
Arf-GAP wi	ACAP1	0.6599	0.01321	Down	81.535	16.5	8	8
DNA polym	POLD3	1.8312	0.013923	Up	51.4	11.2	4	4
WD repeat-c	WDR43	1.8413	0.000582	Up	74.89	16.7	7	7
Protein disu	PDIA6	0.7016	0.0112968	Down	48.121	40	16	16
Platelet-acti	PAFAH1B3	1.4393	0.0329052	Up	25.734	72.7	11	10
Interferon re	IRF4	0.3695	0.0113592	Down	51.772	25.7	9	9
Protein trans	SEC23B	0.7451	0.0402687	Down	86.478	50.7	30	27
Tumor necro	TRADD	0.6475	0.0235521	Down	34.247	40.1	9	9
Kynureninas	KYNU	0.4408	0.0477362	Down	52.351	32.9	13	13
Phosphoeno	PCK2	1.5241	0.0123039	Up	70.698	50	25	24
GDP-Man:M	ALG11	0.6686	0.0127657	Down	55.65	18.1	7	7
Fibronectin	FNDC3B	0.4672	0.0048685	Down	132.89	22.6	18	18
RRP12-like	RRP12	1.7314	0.0193789	Up	143.7	21.3	18	18
Cytochrome	COA6	1.3397	0.0189704	Up	14.116	40	6	6
Cytochrome	COX20	1.4896	0.0300768	Up	13.291	52.5	5	5
Phytanoyl-C	PHYHD1	0.5048	0.0025018	Down	32.41	26.5	6	5
Queuosine s	C9orf64	0.6733	0.025596	Down	39.028	31.4	8	8
Protein wntl	WLS	1.3453	0.0145775	Up	62.253	11.3	5	5
Rootletin OS	CROCC	0.5783	0.0076745	Down	228.52	0.8	2	2
Complex III	LYRM7	2.081	0.0207605	Up	11.955	37.5	3	3
Regulation c	RPRD2	1.7177	0.0341955	Up	156.02	10.3	10	10
Single Ig IL	SIGIRR	1.8146	0.0261148	Up	45.679	21	6	6
Calcium-bin	SLC25A24	1.385	0.0437709	Up	53.354	53.7	25	25
Transmembr	TMEM214	0.7429	0.0467788	Down	77.15	28	14	14
Putative dec	TATDN1	1.7	0.0224163	Up	33.601	37	9	9
Metal transp	CNNM4	1.6402	0.0208638	Up	86.606	16.5	9	6
Acylpyruvas	FAHD1	1.7757	0.0055289	Up	24.843	61.2	10	10
Aspartate--t	DARS2	1.5287	0.0137328	Up	73.562	42.2	21	21
Zinc transpo	SLC30A9	0.7151	0.0232596	Down	63.514	13	6	6
Dehydrogen	DHRS11	3.0327	0.0351046	Up	28.308	51.9	11	11
Olfactomedi	OLFM4	4.8536	0.0229993	Up	57.279	53.1	27	27
Methyltrans	METTL7B	4.2584	0.021775	Up	27.775	42.2	9	9
Protein disu	CRELD2	0.5336	0.0044194	Down	38.191	26.9	8	8
Mucin-6 OS	MUC6	0.3741	0.0449331	Down	257.05	43.2	76	76
Rab11 famil	RAB11FIP1	1.7493	0.0159847	Up	137.17	4.9	5	4
GRB10-inte	GIGYF2	0.6828	0.0329668	Down	150.07	12	10	10
Staphylococ	SND1	0.7531	0.0141955	Down	102	64.8	52	52
Cytochrome	COX15	1.8598	0.026228	Up	46.03	13.2	4	4
tRNA methy	TRMT10C	1.6447	0.0079534	Up	47.346	46.4	16	16
Mitochondri	MAVS	1.4528	0.038277	Up	56.527	29.3	9	9
Wings apart	WAPL	1.6082	0.0037613	Up	132.94	16.5	13	13
Interferon re	IRF2BP2	1.4102	0.0055759	Up	61.024	40.9	14	10
Arpin OS=H	ARPIN	1.4183	0.0380391	Up	24.943	23.9	5	5
E3 ubiquitin	HUWE1	1.4067	0.0006973	Up	481.89	26.8	86	86
Centromere	CENPV	1.5487	0.0407026	Up	29.946	24.4	5	5
E3 ubiquitin	SYVN1	0.6837	0.0061937	Down	67.684	9.7	7	7
Polyadenyla	PABPN1	1.3112	0.0259851	Up	32.749	32.7	8	8
Kinectin OS	KTN1	0.6989	0.0427907	Down	156.27	51.8	65	65
THO compl	ALYREF	1.4433	0.029035	Up	26.888	31.1	6	6

Histone-argi	CARM1	1.6012	0.0177839	Up	65.853	18.4	10	10
Interferon re	IRF2BP1	1.5881	0.0078219	Up	61.687	17.8	8	7
5'-3' exonuc	PLD3	0.5397	0.0006467	Down	54.705	18.6	8	8
WD repeat-c	WDR75	2.3635	0.0166686	Up	94.498	15.1	9	9
Mitofusin-1	MFN1	1.3755	0.0407444	Up	84.159	8.9	5	5
PHD finger	PHF6	2.0034	0.0226105	Up	41.29	31.8	10	10
DnaJ homol	DNAJC10	0.7299	0.0203198	Down	91.079	26.9	17	17
ATP-depenc	SUPV3L1	2.452	0.0135597	Up	87.99	22.5	14	14
Proline-, glu	PELP1	1.7242	0.0014946	Up	119.7	22	14	13
Spartin OS=	SPART	1.484	0.0095928	Up	72.832	12	6	6
Cell cycle ar	CCAR2	1.3929	0.0025325	Up	102.9	39.4	24	24
Cap-specific	CMTR1	1.8995	0.0044533	Up	95.32	11.9	8	8
Lysophosph	ABHD12	1.4522	0.0144391	Up	45.096	43.7	13	13
Probable hy	PNKD	2.3555	0.034879	Up	42.875	20.5	4	4
Actin filame	AFAP1L2	1.8004	0.0386294	Up	91.299	12	5	5
Thioredoxin	TXNDC5	0.4943	0.0002703	Down	47.628	69	27	27
Pyruvate del	PDPR	1.362	0.0408537	Up	99.363	30.7	22	22
DCC-interac	APPL2	1.6934	0.0155995	Up	74.493	39.2	18	17
FAD syntha	FLAD1	1.533	0.0147838	Up	65.265	25.2	10	10
Torsin-1A-ii	TOR1AIP2	1.4397	0.0067693	Up	51.263	33.8	11	9
Protein ABH	ABHD11	1.8969	0.0229724	Up	34.69	41	9	9
Dolichyl-dip	STT3B	0.6453	0.0037175	Down	93.673	18.8	16	15
Polyribonuc	PNPT1	1.4663	0.0074838	Up	85.95	37.8	23	23
Epidermal g	EPS8L1	0.4323	0.0137331	Down	80.25	39	23	22
PDZ domain	GIPC2	2.0652	0.0058775	Up	34.354	30.8	9	9
Nucleolar co	NOC3L	2.0455	0.0012937	Up	92.547	4.4	3	3
Marginal zo	MZB1	0.2847	0.0001499	Down	20.694	71.4	10	10
Protein THE	THEM6	2.0589	0.0058031	Up	23.865	51.9	10	10
Selenoprote	SELENOM	0.6234	0.0349177	Down	16.231	40.7	6	6
Negative elo	NELFB	1.746	0.0447805	Up	65.697	29.5	13	13
Acyl-coenzy	ACOT11	1.4122	0.0353356	Up	68.491	24.7	9	9
Proteasome	PSMF1	1.6108	0.0184043	Up	29.816	14.8	4	4
Engulfment	ELMO1	0.6964	0.0447622	Down	83.829	25.2	15	12
Heat shock p	HSPH1	1.976	0.0147153	Up	96.864	60.8	43	39
CCR4-NOT	CNOT9	1.3483	0.0139027	Up	33.631	36.8	8	8
Dedicator of	DOCK2	0.5867	0.0107731	Down	211.95	25.2	38	38
GPI-anchor	PIGK	1.4275	0.0271625	Up	45.251	23.8	7	7
A-kinase an	AKAP1	2.1325	0.0029762	Up	97.34	28.6	19	19
Geranylgera	RABGGTA	0.681	0.0494265	Down	65.071	33.7	16	16
Disks large	DLG3	1.5448	0.0477045	Up	90.313	17.5	12	9
TATA-bindi	TAF15	1.3582	0.0282362	Up	61.829	17.9	11	9
Glomulin O	GLMN	1.8078	0.0229913	Up	68.207	11.6	6	6
Myeloid-der	MYDGF	0.6531	0.0052543	Down	18.795	37	6	6
Endoplasmic	ERGIC1	0.6584	0.0154044	Down	32.592	48.6	11	11
FAST kinase	TBRG4	3.0376	0.0256276	Up	70.737	30.1	12	12
Interferon-st	ISG20	0.5183	0.001002	Down	20.363	59.1	9	9
Protein FAM	FAM3D	0.3847	0.0233415	Down	24.963	43.3	9	9
Cytochrome	COA7	2.2066	0.0045779	Up	25.709	23.4	3	3
BTB/POZ d	KCTD12	1.4937	0.0239463	Up	35.7	53.8	16	16
60S ribosom	NMD3	1.444	0.0419826	Up	57.603	37.8	13	13
Endoplasmic	ERLEC1	0.7463	0.0252972	Down	54.858	38.1	13	13
Glucosamin	GNPNAT1	0.6955	0.0117918	Down	20.749	64.7	8	8

Ceramide sy	CERS2	1.3389	0.0288173	Up	44.876	11.8	3	3
Methylthiori	APIP	0.5542	0.0072264	Down	27.125	26	4	4
Engulfment	ELMO2	1.4751	0.0032233	Up	82.614	23.5	12	9
PDZ and LI	PDLIM2	1.4344	0.031606	Up	37.458	16.8	4	4
Cleft lip and	CLPTM1L	0.7319	0.0308334	Down	62.228	23.2	11	11
DnaJ homol	DNAJC1	0.5072	0.0141449	Down	63.882	31.4	13	13
Protein LRA	LRATD2	1.4659	0.0100291	Up	34.474	31.3	6	6
Pseudouridy	PUS7	2.8872	0.0246953	Up	75.035	13.8	7	7
Remodeling	RSF1	31.4317	0.007058	Up	163.82	16.3	17	17
Succinate de	SDHC	1.3751	0.0366825	Up	18.61	38.5	4	4
Calcium and	CIB1	1.8479	0.04195	Up	21.703	52.9	9	9
BAG family	BAG1	1.395	0.0297718	Up	38.778	37.4	11	11
NAD-cappe	NUDT12	1.9918	0.0080929	Up	52.075	29	10	10
Ubiquinol-c	UQCC2	1.9478	0.0014194	Up	14.875	32.5	4	4
Agmatinase,	AGMAT	2.604	0.0034798	Up	37.66	40.1	12	12
Protein YIP	YIPF4	0.6089	0.016692	Down	27.082	12.7	3	3
Death-induc	DIDO1	2.5592	0.033479	Up	243.87	7.9	11	11
Dynactin sul	DCTN5	0.6368	0.0048497	Down	20.126	11	2	2
Mini-chrom	MCMBP	2.146	0.0120829	Up	72.979	21.5	8	8
Oxidoreduct	HTATIP2	1.4481	0.0318455	Up	27.049	56.6	14	14
Transmemb	TMED9	0.7225	0.0019449	Down	27.277	46	13	11
Intraflagella	IFT27	0.6239	0.004329	Down	20.48	33.9	5	5
Chitinase dc	CHID1	0.6922	0.016776	Down	44.94	55	16	16
Oxysterol-bi	OSBPL1A	1.7855	0.0052215	Up	108.47	9.5	6	6
Inosine tripl	ITPA	1.6416	0.0417345	Up	21.445	43.3	6	6
Replication	RTF2	1.8637	0.0464137	Up	33.886	18	4	4
Signal pepti	SEC11C	0.4921	0.0077344	Down	21.542	35.9	8	8
Alpha-(1,6)-	FUT8	0.578	0.0021444	Down	66.515	41.6	19	19
Regeneratin	REG4	10.0952	0.0191675	Up	18.23	62.7	11	11
F-box-like/V	TBL1XR1	1.4037	0.0285531	Up	55.594	39.9	15	15
Normal muc	NMES1	4.3833	0.0060784	Up	9.6172	61.4	8	8
(E3-indepen	UBE2O	0.7643	0.0440594	Down	141.29	16.8	14	14
Derlin-2 OS	DERL2	0.3568	0.0098884	Down	27.567	20.5	5	4
Protein FAM	FAM107B	0.7157	0.0119324	Down	15.558	46.6	5	5
Toll-interact	TOLLIP	0.6868	0.0096987	Down	30.281	39.8	7	7
Magnesium	MAGT1	0.6568	0.0216902	Down	38.036	20.9	8	8
Iron-sulfur c	ISCU	1.7648	0.0471578	Up	17.999	23.4	3	3
Phosducin-l	PDCL3	1.562	0.0107326	Up	27.614	25.9	5	5
Golgi reside	ACBD3	0.6597	0.0126412	Down	60.593	41.9	18	18
Mucin-13 O	MUC13	3.2117	0.0273929	Up	54.603	25.6	10	10
Differentiall	DEF6	0.4713	3.375E-05	Down	73.91	28.4	15	14
Golgi-associ	GLIPR2	0.677	0.0203318	Down	17.218	61.7	7	7
Coiled-coil (	CCDC134	1.6272	0.0275262	Up	26.56	19.2	3	3
dCTP pyrop	DCTPP1	2.1138	0.0362684	Up	18.681	50.6	8	8
Mth938 don	AAMDC	2.1377	0.0265686	Up	13.332	57.4	6	6
Prostaglandi	PTGES2	1.4084	0.0423579	Up	41.943	49.1	13	13
Pleckstrin h	PLEKHF2	1.7753	0.0018384	Up	27.797	41.8	6	6
Probable cy	CARS2	1.6493	0.0218474	Up	62.223	14	6	6
Regulator of	UPF2	1.4701	0.0193796	Up	147.81	11.7	10	10
Exportin-5 (	XPO5	2.017	0.0429286	Up	136.31	16.8	16	16
Calcyclin-bi	CACYBP	1.5221	0.0195452	Up	26.21	71.9	18	18
Stromal cell	SDF2L1	0.5409	0.0002994	Down	23.598	63.8	10	10

Vacuolar pr	VTA1	1.4918	0.0248982	Up	33.879	36.8	11	11
Transcriptio	ENY2	1.3982	0.0229416	Up	11.528	36.6	4	4
Scavenger r	CD163L1	0.3338	0.0115721	Down	159.24	28.4	24	24
Sialic acid s	NANS	0.7455	0.0091821	Down	40.307	65.7	18	18
Phosphoribo	PRTFDC1	1.8294	0.0447973	Up	25.673	37.3	6	6
DNA dC->d	APOBEC3C	0.567	0.0282299	Down	22.826	14.2	2	2
Glycoprotei	C1GALT1	0.4871	0.014392	Down	42.202	34.7	9	9
Sister chron	PDS5B	1.3086	0.0491956	Up	164.67	26.8	29	26
1-acyl-sn-gl	AGPAT5	1.9475	0.0088449	Up	42.072	26.4	7	7
Inactive ubi	OTULINL	1.7416	0.0138626	Up	42.195	15.7	5	5
Nucleotide t	NUDT15	1.3445	0.0424275	Up	18.609	43.9	6	6
ADP-ribosy	ARL8B	1.3198	0.0369232	Up	21.539	45.2	9	3
Poly(A) RN	MTPAP	2.1486	0.0158035	Up	66.171	18.4	7	7
PIH1 domai	PIH1D1	2.1673	0.0006123	Up	32.363	18.3	3	3
Probable tR	THG1L	1.7558	0.0333413	Up	34.83	24.2	5	5
E3 ubiquitin	MARCHF5	1.5144	0.0253682	Up	31.231	40.3	8	8
Glutaminyl-	QPCTL	0.5378	0.0004155	Down	42.924	15.4	5	5
Peptidyl-pro	FKBP11	0.7222	0.0274227	Down	22.18	23.4	5	5
Glycerophos	GDE1	1.4263	0.014703	Up	37.718	7.6	2	2
Charged mu	CHMP5	1.7313	0.0337268	Up	24.57	25.1	3	3
Ribosome-b	RRBP1	0.7654	0.0364893	Down	152.45	66.5	70	68
RNA-bindin	RBM27	1.7422	0.018217	Up	118.72	8.2	7	6
Unconventio	MYO1A	2.0063	0.0263142	Up	118.4	42.7	37	34
Set1/Ash2 h	ASH2L	1.9905	0.0043579	Up	68.722	20.7	9	9
Histone dea	HDAC6	1.7812	0.0012868	Up	131.42	7.2	5	5
DnaJ homol	DNAJB11	0.6285	0.0072011	Down	40.513	33.8	13	13
Mitochondri	SLC25A10	1.3357	0.0321992	Up	31.282	36.2	7	7
STE20/SPS	STK39	0.6995	0.0112118	Down	59.473	29.9	13	9
Tryptophan-	WARS2	1.6003	0.0419212	Up	40.146	10.3	3	3
Cysteine and	CHORDC1	1.751	0.02929	Up	37.489	60.5	15	15
F-box only p	FBXO2	6.4748	0.0058157	Up	33.327	28	6	6
REST corep	RCOR1	1.66	0.0153205	Up	53.327	28.5	9	9
General tran	GTF3C4	1.7115	0.0144288	Up	91.981	16.1	10	10
Endothelial	PROCR	1.5622	0.0315353	Up	26.671	19.7	4	4
AP-3 compl	AP3M1	0.6643	0.0002805	Down	46.939	54.8	16	13
Guanine dea	GDA	3.7013	0.0402819	Up	51.002	42.7	15	15
WW domain	WBP11	1.6716	0.0387576	Up	69.997	16.5	9	9
ARF GTPas	GIT1	1.4377	0.0400761	Up	84.34	13.7	8	6
Tyrosine--tR	YARS2	1.5642	0.0002784	Up	53.198	40.5	11	11
Ubiquitin-co	UBE2J1	0.5106	0.007431	Down	35.198	42.8	11	11
Ubiquitin-fo	UFC1	0.6583	0.0283895	Down	19.458	34.7	7	7
Mitochondri	PAM16	1.5507	0.0061829	Up	13.825	30.4	3	3
V-type proto	ATP6V0A2	0.6408	0.0143063	Down	98.081	15.8	10	9
Transformat	TRRAP	1.5325	0.0244771	Up	437.6	5.5	14	14
Transducin	TBL2	0.7254	0.0035681	Down	49.797	51.7	21	21
AFG3-like p	AFG3L2	1.5549	0.0191653	Up	88.583	48.2	35	35
Mitochondri	TIMM13	1.7172	0.0080368	Up	10.5	78.9	7	7
Mannose-1-	GMPPB	0.6868	0.0058587	Down	39.834	42.2	11	11
Immediate e	IER3IP1	0.5876	0.0079996	Down	8.9687	34.1	2	2
Basic leucin	BZW2	1.3502	0.0455829	Up	48.162	26.7	11	10
Harmonin C	USH1C	2.6231	0.0253453	Up	62.21	16.8	7	7
